# Study of the Properties of Blended Cements Containing Various Types of Slag Cements and Limestone Powder

**DOI:** 10.3390/ma14206072

**Published:** 2021-10-14

**Authors:** Małgorzata Gołaszewska, Zbigniew Giergiczny

**Affiliations:** Faculty of Civil Engineering, Silesian University of Technology, ul. Akademicka 5, 44-100 Gliwice, Poland; zbigniew.giergiczny@polsl.pl

**Keywords:** cement composites, limestone cement, heat of hydration

## Abstract

It is currently vital to use more environmentally friendly cementitious composites, such as blended slag-limestone cements. However, many properties of slag-limestone cements are not yet fully research, especially in regards to the effect of limestone properties on properties of mortars and concrete. In the research, three types of slag cements were mixed with two types of limestone to obtain multi-component slag-limestone cements. Tests of rheological properties, heat of hydration, and compressive strength were conducted to ascertain the effect of limestone on the cement properties and to check the viability of this type of cement for engineering practice. It was found that the addition of up to 10% of limestone to slag cements did not have negative effects on tested properties; however, the exact influence of limestone was dependent on limestone particle size distribution. Increasing the amount of limestone in limestone-slag cements to 15% significantly decreased the compressive strength of the mortars and decreased hydration heat but had no significant effect on rheological properties.

## 1. Introduction

Sustainable development policy poses an ongoing challenge for the cement industry due to the harsh restrictions on CO_2_ emissions. Currently, cement production is responsible for 5–8% of all anthropogenic CO_2_ emissions, and available data indicate that this percentage will increase in the next few years [1]. High CO_2_ emissions in clinker production are a result of a process of chemical breakdown of raw materials, mainly calcium carbonate CaCO_3_, during the clinker firing process and the high energy demand for this reaction to occur, as the temperature in the cement kiln reaches over 1450 °C [2]. Currently, the production of 1 t of clinker is responsible for the emission of between 0.825 and 0.890 t of CO_2_, in which at least 0.6 t is a direct result of the reaction of calcium carbonate chemical breakdown [3,4,5].

While there is research conducted into alternative methods of clinker production, for example, by Ellis et al. [6], the most efficient and economically sound method of lowering the negative impact of clinker production is to use waste materials [7], make the more efficient use of cement in concrete and mortar production, and increase the substitution of clinker by supplementary cementitious materials [8]. The European Standardization Committee has begun to broaden the range of common cements in the working draft of standard prEN-197-5 [9] by extending the possibilities of using non-clinker main components in cements by introducing CEM II/C-M and CEM VI, which will include, respectively, up to 50 and 65% of supplementary cementitious materials. New types of common cements heavily use the addition of limestone to cement in amounts from 6 to 20%.

Limestone is one of the main non-clinker components of cement in the countries that are members of Cembureau [10] due to its high accessibility for all cement plants and positive influence on cement hydration [11]. While limestone itself is mostly inert as the main constituent of cement [12], its physical properties can enhance the performance of cement composites. Firstly, limestone is softer than clinker, and thus it is easier to obtain a higher specific surface area [13]. This, in turn, allows the limestone to act as a microfiller reducing porosity, increasing strength in the initial period of hardening and improving workability, and reducing the drainage of water from a concrete mix (“bleeding”), as was proven by Dhir et al. [14], Githachuri and Alexander [15], Moir et al. [16] and Ramezanianpour et al. [17].

Slag-limestone cements have been the subject of research, which indicates that there might be a possibility of the synergistic effect of limestone and slag [18,19,20]. Tests conducted by Ramezanianpour and Hooton [21] found that at low contents of granulated blast furnace slag, content up 20% of limestone, no significant reduction in concrete compressive strength is observed. In the case of a higher content of granulated blast furnace slag, approximately 30–50% of cement mass, a decrease in the beneficial effect of limestone, and deterioration of 28-day strength were observed. Adu-Amankwah et al. [22] and Kucharczyk et al. [23] found that slag-limestone cements are characterized by a slightly accelerated hydration process and higher heat released in the initial hardening period, as well as similar or even higher total hydration heat in relation to Portland cements.

Aside from the physical effects on the hydration process, calcium carbonate reacts with reactive aluminates from Portland cement clinker [12]. This leads to the formation of calcium hemi- and mono-carboaluminate hydrates and the stabilization of ettringite, which can enhance the compressive strength [21]. Moreover, the fact that calcite reacts with C_3_A to form carbonaluminates makes it possible for CaCO_3_ to act to a limited degree as a regulator of the setting time [24,25,26]. This synergic effect has been observed by De Weerdt et al. [27,28] and Bentz et al. [29], also in the case of multi-component cements with fly ash and limestone. In the case of cements with blast furnace slag and limestone, the reaction of calcite with reactive alumina can also be observed, but due to additional calcium sulfate, the effect is dependent on the alumina [30].

The current state of knowledge about lime-slag cements does not, however, allow for unequivocal determination of how the content of limestone and blast furnace slag affects the rheological properties of mortars and concretes. Courard and Michel [31] assessed the workability of concretes made of lime-slag cements by testing the consistency with the concrete slump test. In these tests, the content of limestone did not have a significant impact on consistency, and the addition of slag slightly worsened the consistency (workability).

There are three ways of obtaining slag-limestone cements:

- Inter-grinding the constituents;
- Designing the composition of cements from separately ground ingredients; and
- Introducing limestones as a constituent of concrete (additive type I according to EN 206 [32]).

The first two methods are available only to cement plants, while the last method of obtaining slag-limestone cements is also often used in concrete plants and thus may prove to be more universal.

Therefore, the presented research aims to test the basic properties of the slag-limestone cements obtained by mixing easily obtainable industrial Portland slag cements CEM II/A, B-S, and slag cement CEM III/A with ground limestone in amounts of 5, 10, and 15%. There were two types of limestone used to better see the influence of the limestone characteristic on the properties of slag-limestone cements. In the course of the research, conducted were rheological tests of yield stress and plastic viscosity after 5 and 60 min from the moment of mixing all the ingredients, compressive and flexural strength after 2, 7, 28, and 90 days of curing, and heat of hydration in the first 72 h of the reaction.

## 2. Materials and Methods

### 2.1. Methods

The tests were performed mostly on mortars, with the exception of the heat of hydration test, which was performed on cement paste. The composition of all mortars used in the research was based on a standard mortar composition (450 g of cement, 225 g of water, 1350 g of standard sand) and preparation, as described in EN 196-1 [33]. The mixing procedure was conducted in an automatic mortar mixer and lasted 3.5 min: 30 s of slow mixing of cement with water, 30 s of slow mixing while adding standard sand, 30 s of fast mixing, 90 s pause, and after that 30 s of fast mixing. In the case of rheological measurements, the w/c ratio of all of the mortars was increased from 0.5 to 0.55, as the stiffness of mortars with w/c = 0.5 was too high for the apparatus to make the measurement, and therefore it was impossible to perform the test.

**Figure 1:**
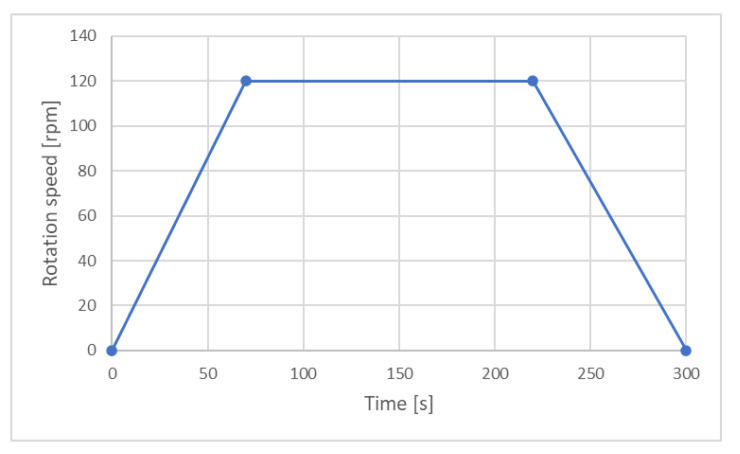
Rheological measurement procedure.

The rheological tests were performed in rheometer Viscomat NT (Schleibinger, Buchbach, Germany) with fishbone probe, and simplified Bingham’s model was applied to calculate the values of rheological parameters:

(1) M=g+hN

where: *M*-shear resistance moment, *g*-shear resistance, *h*-viscous flow resistance, *N*-rotational speed. In this equation, the flow limit τ_0_ corresponds to the shear resistance g, and the plastic viscosity η_pl_-the viscous flow resistance h. The sample was put in the apparatus immediately after mixing. The single test lasted 5 min, and its exact course is shown in Figure 1. Data for the Bingham equation were collected during the slowing of the rotational speed so that any technical issues with measurement start do not affect the measurement. After the test, the sample from the apparatus was put back into the mortar container. After 60 min of mixing, the mortar was mixed for 15 s at low speed in a mixer to allow it to be placed back in the apparatus. Then the 5-min measuring cycle was repeated. In-depth descriptions of rheological measurement procedures can be found in [34]. Each measurement was performed three times.

The compressive strength of mortars was tested after 2, 7, 28, and 90 days of curing. The preparation of samples and the testing process were performed according to the standard EN-196-1 [33]. After mixing, the mortar was compacted in two layers into the forms, into samples 40 × 40 × 160 mm. After 24 h, the forms were removed, and samples were cured in water of temperature 20 °C. The samples were tested in controls PILOT automatic compression tester (Controls, Warsaw, Poland). For each measurement, 6 samples were prepared and tested.

The heat of hydration was measured by the isothermal calorimetry method in TAM air isothermal calorimeter (TA Instruments, New Castle, UK) on cement paste for 72 h from the moment of adding water to cement. The measurement was conducted according to standard [35], at a temperature of 20 °C. The sample of 5 g cement was mixed with 2.5 g of water in the chamber of the calorimeter, which allowed us to measure the heat of hydration from the very beginning of the hydration process.

### 2.2. Materials

To obtain the slag-limestone cements, three types of commercially available slag cements: CEM II/A-S 52.5N, CEM II/B-S 32.5R, and CEM III/A 42.5N, were mixed with two types of limestone, labeled T and B.

**Figure 2:**
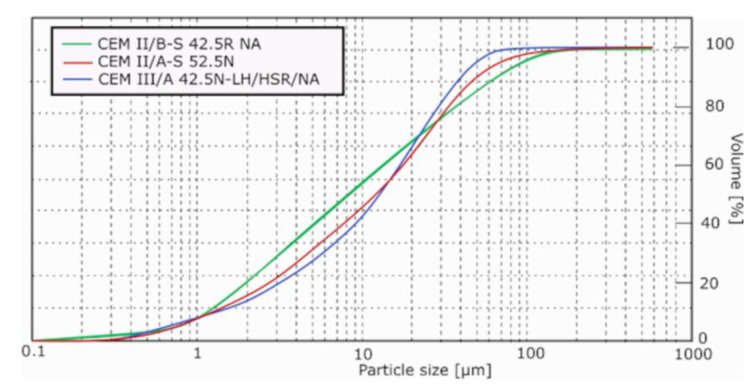
Particle size distribution of slag cements used in the research.

The chemical and phase composition of the slag cements is presented in Table 1 and Table 2, and the determined standard properties of the cements are shown in Table 3. The cements are commercially available, and their composition was not made to specification; however, information about the exact amounts of slag in their composition was obtained from the producer. The particle size distribution of the cements is presented in Figure 2.

**Figure 3:**
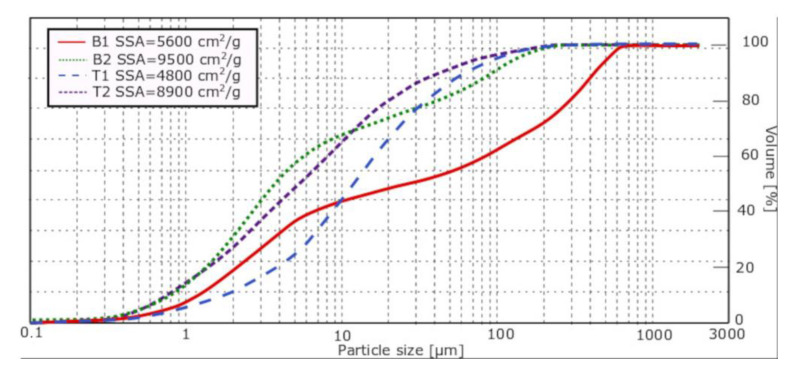
Particle size distribution of limestone used in the research.

**Figure 4:**
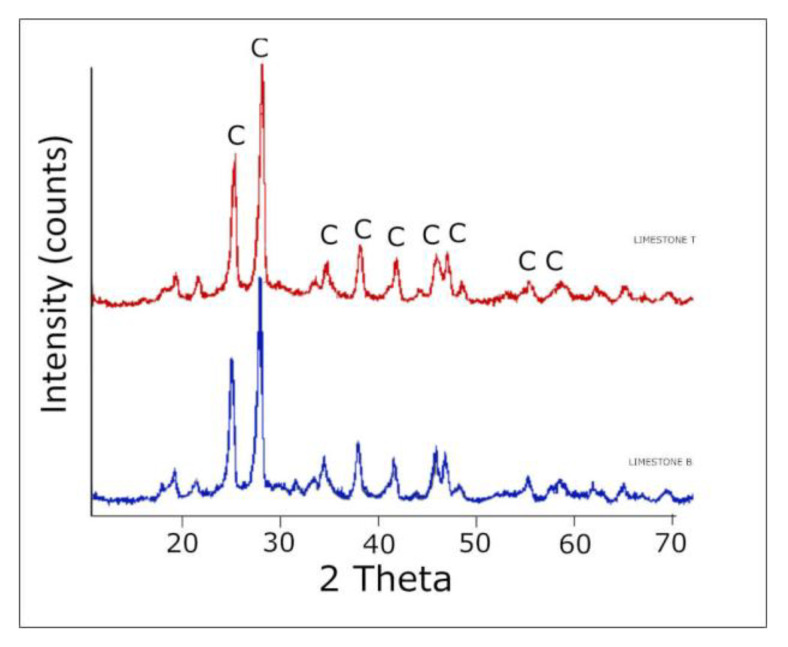
XRD tests of the limestones—limestone T (red line) and B (blue line).

Two types of limestone were used: limestone B and limestone T. Their chemical composition is presented in Table 4, particle size distribution in Figure 3. The chemical composition of the limestones fulfills the requirements for limestone used in the cement, set by standard EN 197-1 [36], namely, that content of CaCO_3_ is higher than 75%. XRD (Malvern Panalytical, Malvern, UK) of the limestones, presented in Figure 4, shows that the limestones consist mostly of calcite.

Limestone T and B come from different quarries and different geological formations. Limestone B comes from Jurassic strata and limestone T from Triassic strata. Both limestones were ground in a ball mill to two specific surface areas, around 5000 cm^2^/g (marked as “1”) and around 9000 cm^2^/g (marked as “2”). The actual specific surface area of all limestones is presented in Figure 3. The issue of the mineralogy of limestone was raised by Damineli et al. [37]. Previous testing of the influence of fineness of T and B limestones on properties of limestone cements and slag-limestone cements has shown no effect of increased fineness on rheological properties of hydration heat; however, its effect on compressive strength was ambiguous [38,39]. Therefore, high fineness limestone was used only in the compressive strength tests.

The proportions of the slag-limestone cements used for the research are presented in Table 5. The range of limestone addition was set to 5–15%. Previous research [40,41], as well as previous tests by the authors [41], had shown that using more than 15% of limestone can greatly decrease the strength of mortars and concrete. Seeing as the assumption of undertaken research was to create and test cements that could be easily implemented in practice, the highest addition of limestone was set to 15%. The cements were obtained after 30 min of homogenization of slag cements with limestone in a laboratory mixer.

## 3. Results

### 3.1. Rheological Properties

**Figure 5:**
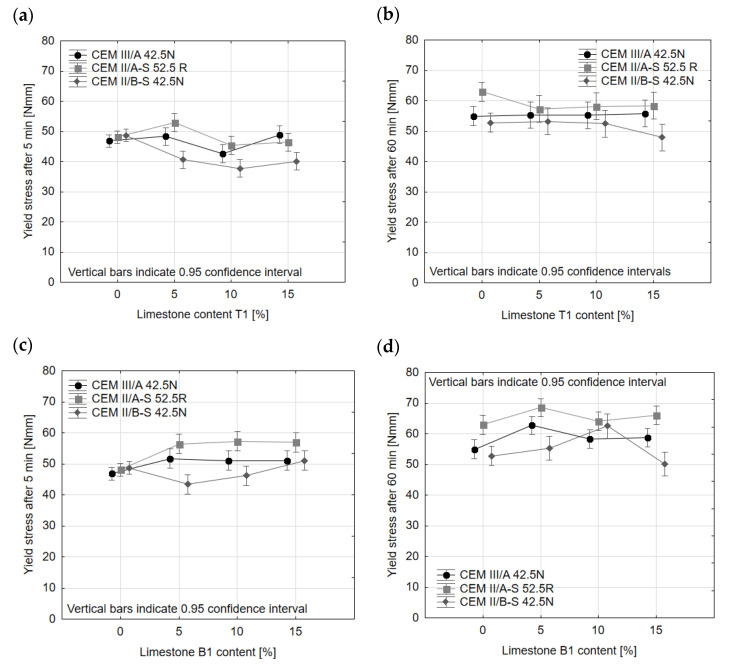
Yield stress of slag-limestone cement after 5 min (**a**,**c**) and 60 min (**b**,**d**) from adding water to cement.

**Figure 6:**
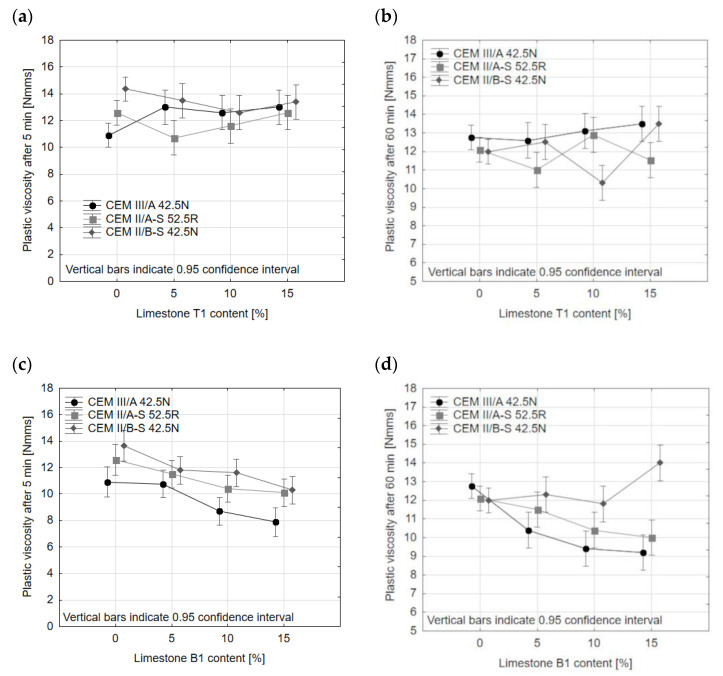
Plastic viscosity of slag-limestone cements after 5 min (**a**,**c**) ad 60 min (**b**,**d**) from the moment of mixing.

The results of rheological tests of multi-component slag-limestone cements obtained by homogenization of Portland slag cements with limestone T1 and B1 are presented in Figure 5 and Figure 6. The tests were conducted on the modified standard mortar (1350 g of standard sand, 450 g of cement) with a w/c ratio changed from 0.5 to 0.55 due to the technical limitation of the rheometer. As it can be seen in Figure 4, the addition of limestone T1 to cement did not change the yield stress of cements CEM II/A-S 52.5N and CEM III/A 42.5N after 5 or 60 min. In the case of CEM II/B-S 42.5N, the yield stress after 5 min from mixing water with cement clearly decreased in the presence of limestone; however, this effect was not present after 60 min, as the yield stress is the same for all CEM II/B-S 42.5N cements with 5–15% of limestone content.

The addition of limestone B1 to cement caused an increase in the yield stress in mortar with cement CEM II/A-S 52.5N and, to a lesser extent, CEM III/A 42.5N. The yield stress of mortar with CEM II/B-S 42.5N with limestone B1 decreased slightly for 5% addition of limestone, but for the addition of 10–15% remained similar to the yield stress of reference mortar. The effect of the addition of limestone B1 on yield stress after 60 min of mixing is difficult to gauge due to the great variability of the obtained results. However, a general trend of increase in yield stress can be noticed for all types of cement.

The results indicate that the yield stress is dependent on the type of limestone used. This effect could be connected with the particle size distribution of the limestone. Limestone B is characterized by a discontinuous distribution of grains and a high content of coarse grains, which can be detrimental to yield stress. Limestone T, being of a continuous particle size distribution and having less-coarse grains, did not influence the yield stress in a negative way, even decreasing it in the case of cement CEM II/B. It was previously established that in the given range of variability, the specific surface area of limestone does not influence yield stress in a significant way [38,42].

It should be noted that the type or amount of limestone did not affect the loss of workability in time to a significant degree, as the type of cement played a decisive factor. There was no indication that in the range of 5–15% of limestone content, there is a clear trend linking the amount of limestone to the change in yield stress. The yield stress of mortars with CEM II/A-S 52.5N increased by 31% in the first 60 min after mixing, while yield stress of mortars with CEM II/A-S 52.5N with limestone increased by 20% on average, with the lowest increase of 8% for the addition of 5% of limestone T1, and highest increase of 29% for addition of 10% of limestone T1. For mortars with cement CEM III/A 42.5N, there was no notable change in the loss of consistency, with the 17% increase in yield stress in 60 min from mixing of reference mortar and average yield stress increase for mortars with limestone being 18%. Only in the case of mortars with CEM II/B-S 42.5N, the addition of limestone T1 and B1 could cause an increase in yield stress after 60 min of mixing. For reference mortars, the yield stress increased by 18% during the first 60 min of mixing, while in the case of mortars with CEM II/B-S 42.5N and limestone, the average increase in yield stress was 29%.

In the case of plastic viscosity, the results of which are presented in Figure 3, the effect of both limestone T1 and limestone B1 mirrors the effect of limestones B and T on yield stress. Limestone T added in an amount of 5–15% of cement mass does not change the plastic viscosity of mortars after both 5 and 60 min, while with the increase in B limestone addition, the plastic viscosity decreases.

This, similarly to the results of yield stress measurements, can be attributed to the difference in the particle size distribution of limestone.

The addition of limestone to cement has an effect on the increase in plastic viscosity during 60 min from adding water to cement. In the case of mortars with CEM II/A-S 52.5N, there was no increase in plastic viscosity during the first 60 min of hydration, both in the case of reference mortar and mortars with slag-limestone cements. However, for reference slag cements with high content of ground granulated blast furnace slag, the plastic viscosity increased in time: for CEM III/A 42.5N, the increase was 17%, and for CEM II/B-S 42.5N, the increase was 20%. This may be connected to the fact that the cements with high content of ground granulated blast furnace slag are characterized by lower density, meaning that the volume of binder in mortars is higher; this leads to an increased tendency to form a gel structure and intermolecular connections [43].

However, the addition of limestone stops the increase in plastic viscosity, as an average increase in plastic viscosity for mortars with CEM III/A 42.5N and limestone is only 4%, and 0% in the case of CEM II/B-S 42.5N (in this case, however, the spread of results is from −18% to 17%, with the majority of results centered around 0% increase). This effect may be due to the fact that plastic viscosity increase is mostly dependent on the concentration of the clinker grains and their flocculation [44,45]. The filler effect of limestone, which causes the more even distribution of clinker grains, may disperse the clinker grains, preventing their coagulation during the first hour of hydration.

The results indicate that in relation to rheological properties, there might be an underlying issue of compatibility between the cement and limestone. It can be seen that the mortars with CEM II/B-S 42.5N and limestone show signs of quicker workability loss; however, more tests are required to fully present the possible negative interaction between limestone and slag cement. The rheological properties of slag-limestone cements have not been well described in the available literature; however, obtained results show the same relation as tests of consistency conducted by Courard and Michel [31], who also did not observe any changes in concrete consistency with the increase in limestone content in limestone-slag cements.

### 3.2. Heat of Hydration

**Figure 7:**
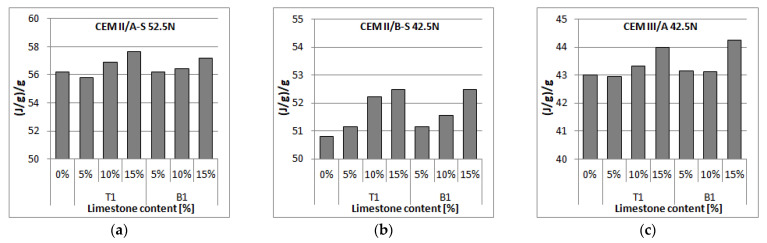
The ratio of the total heat of the slag-limestone cement hydration after 72 h to the weight content of slag cement in multi-component slag-lime cements with (**a**) CEM II/A-S 42.5N, (**b**) CEM II/B-S 42.5N, (**c**) CEM III/A 42.5N cement.

The heat of hydration during the first 72 h of hydration of slag-limestone cements with limestones T1 and B1 is presented in Table 6, while the heat flow is shown in Figure 7.

The addition of limestone to cement reduces the heat of hydration after 72 h. The decrease does not depend on the type of limestone, as the difference between the heat of hydration of slag cements with limestone T1 and B1 is, in every case, less than 0.5%. The range of decrease in the heat of hydration after 72 h is not equal to the amount of clinker that was replaced by limestone. This effect indicates that the presence of limestone has a positive effect on the effectiveness of clinker and/or ground granulated slag hydration. As can be seen in Figure 6, with the increase in limestone content, the effectiveness of slag cement, measured as an amount of heat generated by a gram of slag cement, increases.

Limestone is a mostly inert constituent of cement, with only up to 5% of limestone mass present in cement undergoing a reaction with alumina phases to form calcium carboalumiate [46,47]. While this effect may be a part of the additionally generated heat during hydration, it is more likely, that it is linked to a physical effect limestone has on clinker and ground granulated blast furnace slag. Limestones T1 and B1 are finer than slag cements used in the research, and thus it can pull apart the conglomerating grains of clinker and slag, allowing for better access of water to each and every particle, thus increasing the hydration rate [48]. Moreover, small grains of limestone can act as a nucleation seed, thus speeding up the process of hydration of clinker. For all three slag cements, the increase in slag cement effectiveness is similar, which may lead to a conclusion that the beneficial effect of limestone is not restricted to clinker hydration, but there is also a positive effect on hydration of ground granulated blast furnace slag. The better effectiveness of ground granulated blast furnace slag is connected to the better water access to the slag grains; however, it can also be presumed that it may be the result of the acceleration of the blast furnace slag reaction, caused by a decrease in the availability of aluminates in the presence of limestone [22].

**Figure 8:**
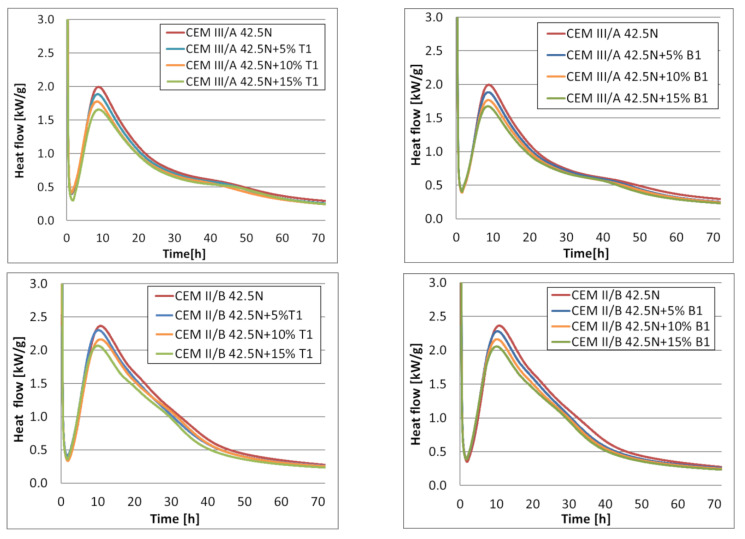

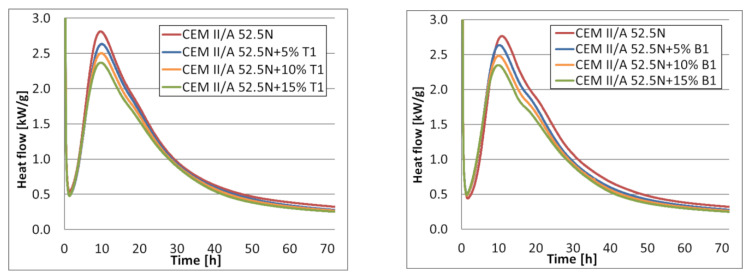
Heat flow of blended cements with limestone T1 and B1.

The effect of limestone on the hydration of slag and clinker is also reflected in the course of the heat flow rate (Figure 8), where it can again be seen that as the limestone content increases, the maximum rate of hydration heat release decreases. A noticeable acceleration of the alite reaction occurs, as indicated by the shortening of the induction phase, which can be seen in Figure 7. Increased hydration rates of cements with limestone addition have also been noted by Zajac et al. [49], Xuan et al. [40], and Puerta-Falla [50].

In the presence of limestone, a significant drop in maximum heat of hydration can be observed, which is linked with the decrease in clinker content. On the other hand, however, there can be observed a shortening of the induction phase. This effect can be attributed to the increased speed of alite reaction in cement in the presence of limestone. This is due to the previously mentioned filler effect and the nucleation seeding of limestone. This effect is most pronounced in the case of CEM II/A-S 42.5N, which is connected to the fact that it has the highest content of Portland clinker.

It should be noted that the presence of limestone also causes an increase in the second heat flow maximum. This effect is the result of an increase in the intensity of the reaction of ettringite formation in the presence of GGBFS [51]. In the presence of limestone, the extrema are more pronounced, especially in the case of cements CEM II/B -S and CEM III/A. It is hard to discretion the exact reason for this effect; however, it might be connected to the higher amount of AFm phase created in the presence of limestone [52], and as well as the acceleration of the reaction of ground granulated blast furnace slag, caused by a decrease in the availability of alumina phases in the presence of limestone, as proven by Adu-Amankwah et al. in [22].

Similar effects were observed by Xuan et al. [40] and Kucharczyk et al. [23], who observed that with the increase in limestone content, the hydration peaks decreased. No mentions of increased heat flow were found in the available literature.

### 3.3. Compressive Strength

**Figure 9:**
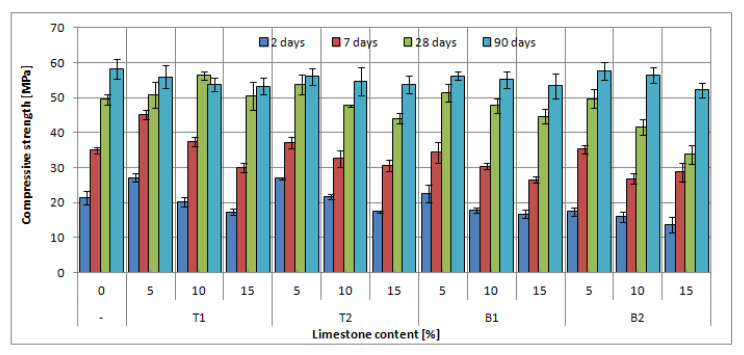
Compressive strength of blended slag-limestone cements made of CEM II/A-S 52.5N.

**Figure 10:**
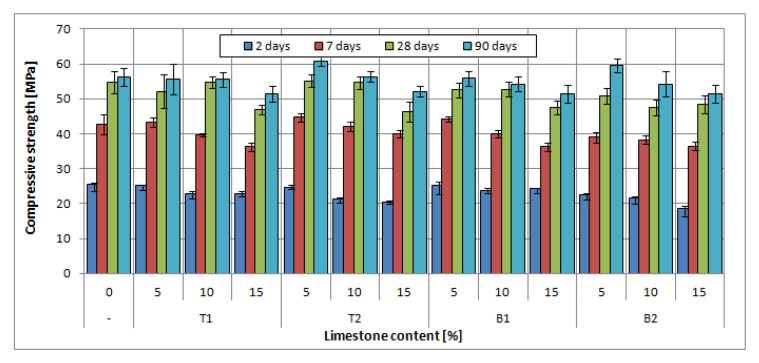
Compressive strength of blended slag-limestone cements made of CEM II/B-S 42.5N.

**Figure 11:**
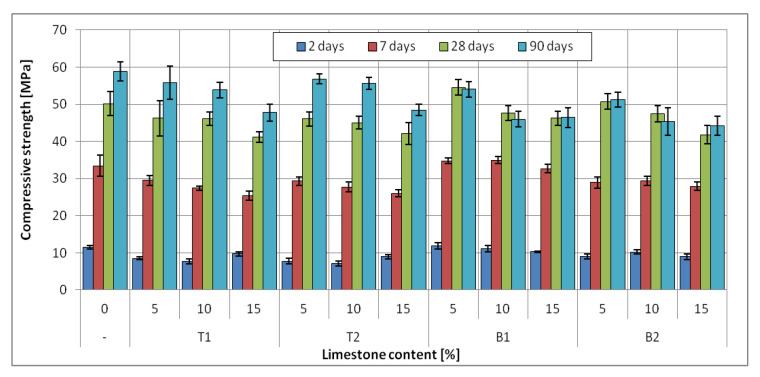
Compressive strength of blended slag-limestone cements made of CEM III/A 42.5N.

The results of the compressive strength test for slag-lime cements are presented in Figure 9, Figure 10 and Figure 11, with a marked standard deviation of obtained results.

The addition of limestone in the amount of 5% of cement mass did not negatively affect the compressive strength of all slag-limestone cements. In some cases, there was an increase in compressive strength after 2 and 7 days, especially visible in the case of CEM II/B-S and CEM II/A-S. The increase was up to 6%. This effect can be linked to the physical effect of limestone on clinker hydration; small limestone grains can act as a nucleation seed, accelerating the clinker hydration [53,54], which could be seen in the results of hydration heat tests (Figure 8). Moreover, limestone prevents the conglomeration of clinker grains, allowing for a higher hydration rate of clinker [55]. With the increased hydration rate in the early stages of hydration, the early compressive strength can also be increased, as the process of structure development is faster. Seeing as there is more clinker content in cements CEM II/A-S and CEM II/B-S, this effect is much more visible than in the case of CEM III/A.

The addition of limestone to slag cement in an amount of 10–15% reduced the strength of cements both in the early (2 and 7 days) and later (28, 90 days) periods. The decrease in average strength at 15% limestone content, i.e., the maximum amount tested for slag cements, is about 10%. The largest decrease in strength, even by 24%, is observed for CEM II B-S. The decrease is, naturally, linked to a lower content of the binder in the slag-limestone cements. In the case of the addition of over 10% of limestone, the effect of clinker dilution is more pronounced than the beneficial effect of limestone on the rate of hydration. However, it should be noted that, in general, the decrease in strength is less than the percentage of limestone. As it has already been noted, limestone is an almost inert component for which the effect on strength is virtually negligible. Slight decreases in strength are, therefore, a sign of the beneficial effect of limestone on the hydration of clinker and granulated blast furnace slag. However, it should be noted that for the tested slag-limestone cements, cements with a content of 5–10% limestone met the strength class requirements for slag cements from which they were prepared. It was not until the 15% limestone content that the cement strength class was decreased.

The type of limestone had an effect on the early compressive strength of mortars with CEM II/A-S and CEM II/B-S. At the two- and seven-day mark, the differences between slag-limestone cements with limestone T and B were up to 50%; however, after 28 days of curing, there was no discernible difference in compressive strength of mortars. The same cannot be said for mortars with cement CEM III/A, in case of which mortars with limestone B were characterized by higher compressive strength in early stages; however, after 28 days, mortars with limestone T have similar or higher (up to 20%) compressive strength than mortars with limestone B.

The specific surface area of limestone did not affect the compressive strength after 28 and 90 days. The difference between the compressive strength of mortars with different specific surfaces did not exceed 5% for both B limestone and T limestone; therefore, the effect could be considered negligible, as the standard deviation was, on average, around 3%. At earlier dates, however, differences of up to 30% in strength can be seen in the case of limestone B1 and B2. Interestingly, the cements with limestone with a lower specific surface area had exhibited consistently higher strength. This effect may be connected to the higher water demand of finer limestone fraction [55,56]. The lower amount of free water in the early days of hydration may negatively influence strength development. This effect, however, requires further consideration.

The compressive strength to hydration heat ratio of the multi-component slag-limestone cements is shown in Figure 9. Hydration heat after 72 h was compared to that after 7 days.

The compressive strength after 2 days is proportional to the hydration heat generated. This relationship is linear but not strict. This may be due to the fact that the analysis of multi-component cements includes three different cements with different strength gain rates and very different hydration heat, which may disturb the results. It should be noted that the relationship between the heat of hydration and compressive strength of mortars was a subject of research by Yoda et al. [57], as well as Baran and Pichniarczyk [58]. The results had shown that while there is a strict relationship between the compressive strength and heat of hydration of Portland cements, the relationship was not as clear in the case of cements with ground granulated blast furnace slag, and this effect may also play a role in the conducted research on multi-component slag-limestone cements. The difference in the rate and the process of ground granulated blast furnace slag hydration may be connected to the uneven distribution of the relationship between the compressive strength and heat of hydration after 2 days from mixing.

## 4. Conclusions

In the presented research, tests of rheological properties, the heat of hydration and compressive strength of mortars with blended slag-limestone cements were obtained by homogenizing commonly available slag cements with two types of limestone T and B in amounts of 5, 10, and 15% of cement mass. Conducted research leads to the following conclusions:

- The effect of limestone addition on the rheological properties of the mortar was dependent on the type of limestone. The addition of up to 15% of continuous-graded limestone to slag cements did not influence yield stress and plastic viscosity in a significant way, while the addition of gap-graded limestone caused an increase in yield stress and decrease in plastic viscosity. This may indicate that the different effects of both limestones on rheological properties may be dependent on the particle size distribution of limestone;
- Introduction of limestone to cement composition reduced the heat of hydration after 72 h. The decrease was dependent on the amount of limestone;
- The introduction of limestone to slag cement increased the heat generated by a gram of slag cement. This effect may be attributed to the nucleation effect of limestone. Fine grains of limestone act as nucleation seeds for clinker, increasing the hydration rate;
- No effect of the particle size distribution of limestone was observed in regards to hydration heat development;
- The substitution of slag cement with limestone in amount up to 10% of cement mass did not negatively affect the compressive strength of all cements; however, a decrease in compressive strength occurred for mortars with 15% of limestone in cement, especially in the case of blended cements based on CEM III/A 42,5N;
- The type of limestone had an effect on the early compressive strength of mortars, with the continuous-graded limestone to slag cements were characterized by higher strength than mortars with gap-graded limestone. This effect was not observed after 28 or 90 days.

Obtained results indicate the possibility of wider use of limestone in the composition of cement and/or concrete with granulated blast furnace slag due to the synergy effect resulting from different properties of these components. To definitively put those types of cements into practical use, more testing is, however, necessary, connected to the compatibility issues of slag and limestone, durability, and properties of concrete with slag-limestone cements.

## Figures and Tables

**Table 1:** Slag cement composition.

Constituent	Amount of Constituent, % Mass in Cement		
	CEM II/A-S 52.5N	CEM II/B-S 32.5R	CEM III/A 42.5N
SiO_2_	22.9	25.2	30.0
Al_2_O_3_	5.4	5.6	6.4
Fe_2_O_3_	2.41	2.13	1.81
CaO	60.3	57.4	52.6
MgO	2.1	2.7	4.1
Na_2_O	0.21	0.23	0.28
K2O	0.74	0.64	0.61
Na_2_O_eq_	0.69	0.65	0.69
SO_3_	2.6	2.6	2.7
Cl^−^	0.073	0.069	0.066

**Table 2:** Slag-limestone cement phase composition.

Constituent		Amount of Constituent, % Mass in Cement		
		CEM II/A-S 52.5N	CEM II/B-S 32.5R	CEM III/A 42.5N
Portland clinker	C_3_S	56.15	47.37	28.4
	C_2_S	5.27	3.97	4.77
	C_3_A	6.54	5.44	3.72
	C_4_AF	5.78	4.87	3.77
Slag		13.4	31.0	51.0

**Table 3:** Properties of the slag cements.

Properties	Cement Type		
	CEM II/A-S 52.5N	CEM II/B-S 42.5N-NA	CEM III/A 42.5N-LH/HSR/NA
Initial setting time (min)	204	201	222
Volume stability (mm)	0.1	0.5	0.3
Specific surface area (cm^2^/g)	4100	4050	4500
Heat of hydration (J/g)	281	254	215
Compressive strength (MPa)			
after 2 days	25.4	21.4	11.6
after 28 days	54.7	49.5	45.1
after 90 days	56.3	58.2	58.9

**Table 4:** Chemical composition of limestones used in the research.

Constituent		LOI	SiO_2_	Al_2_O_3_	Fe_2_O_3_	CaO	MgO	SO_3_	Cl^−^	CaCO_3_ Content
Amount of constituent, % mass in limestone	Limestone B	41.7	4.6	0.7	0.3	52.4	0.7	0.17	0.022	94.8
	Limestone T	42.7	1.4	0.4	0.5	53.2	1.5	0.02	0.007	97

**Table 5:** Composition of slag-limestone cements used in the research.

Cement Type	Slag Cement Content, % Mass	Limestone T, B Content, % Mass
CEM II/A-S 52.5 R	95	5
	90	10
	85	15
CEM II/B-S 42.5N	95	5
	90	10
	85	15
CEM III/A 42.5N	95	5
	90	10
	85	15

**Table 6:** Heat of hydration of slag-limestone cement with limestone T1 and B1.

Cement Type	Limestone Type	Limestone Content (% Mass)	Heat of Hydration (J/g) After						
			1 h	12 h	24 h	36 h	41 h	48 h	72 h
CEM III/A 42.5N	-	0%	14	69	124	157	168	182	215
	T1	5%	14	68	119	150	161	174	204
		10%	14	66	114	144	154	167	195
		15%	11	58	105	134	144	157	187
	B1	5%	15	69	120	152	163	176	205
		10%	13	64	113	143	154	166	194
		15%	14	63	110	139	150	162	188
CEM II/A 52.5 N	-	0%	20	94	178	221	233	247	281
	T1	5%	20	88	169	210	222	235	265
		10%	19	86	163	203	215	227	256
		15%	18	83	156	196	206	218	245
	B1	5%	19	87	170	213	224	237	267
		10%	18	84	162	203	214	226	254
		15%	18	81	155	195	205	216	243
CEM II/B 42.5N	-	0%	12	70	149	198	211	224	254
	T1	5%	13	72	147	192	203	215	243
		10%	11	65	138	184	196	208	235
		15%	12	67	135	177	187	198	223
	B1	5%	13	73	148	192	203	215	243
		10%	13	70	141	184	194	206	232
		15%	12	68	136	177	187	198	223

## Data Availability

The data presented in this study are available on request from the corresponding author.
